# Supplementary material for: Characterization of Cognitive, Language and Adaptive Profiles of Children and Adolescents with Malan Syndrome
Source: J Clin Med. 2022 Jul 14;11(14):4078. doi: 10.3390/jcm11144078 (PMC9316998; doi:10.3390/jcm11144078)
Supplement: Supplementary file 1 [file jcm-11-04078-s001.zip › jcm-1734309-supplementary.pdf]

Supplementary Data File

Supplementary Table S1

Main Malan syndrome clinical features in the present cohort (modified from Macchiaiolo et al 2022)		
Features		Frequency (HPO term)
Facial features	Macrocrania	*OBLIGATE* (HP:0040280)
	Prominent forehead/frontal bossing	*OBLIGATE* (HP:0040280)
	Long/triangular face	*VERY FREQUENT* (HP:0040281)
	Down-slanting palpebral fissures	*FREQUENT* (HP:0040282)
	Short nose/anteverted nares	*FREQUENT* (HP:0040282)
	Everted lower lip	*VERY FREQUENT* (HP:0040281)
	Prominent chin	*VERY FREQUENT* (HP:0040281)
Neurological	ID/DD	*OBLIGATE* (HP:0040280)
	Epilepsy/EEG anomalies	*FREQUENT* (HP:0040282)
	Episodic Ataxia/dizziness and nausea	*OCCASIONAL* (HP:0040283)
Brain MRI abnormalities	WV, HCC, CM1	*FREQUENT* (HP:0040282)
Ophthalmological	Refractive Disorders	*VERY FREQUENT* (HP:0040281)
	Strabismus	*FREQUENT* (HP:0040282)
	Blue sclerae	*FREQUENT* (HP:0040282)
	Optic Nerve Hypoplasia	*OCCASIONAL* (HP:0040283)
Musculoskeletal	Slender Habitus	*OBLIGATE* (HP:0040280)
	Long Hands	*FREQUENT* (HP:0040282)
	Abnormal spine curvatures	*FREQUENT* (HP:0040282)
	Pectus Excavatum/Carinatum	*FREQUENT* (HP:0040282)
	Pes Planus	*FREQUENT* (HP:0040282)
Cardiovascular	Aortic bulb dilatation	*EXCLUDED* (HP:0040285)
	Mitral valve Regurgitation	*FREQUENT* (HP:0040282)

Supp. Table S1 Main Malan syndrome clinical features in the present cohort (modified from Macchiaiolo et al 2022. *J.orphanet of Rare Diseases, accepted:* DOI: 10.1186/s13023-022-02384-9)
Features are reported following HPO criteria for frequencies that records clinical features as OBLIGATE (Always present, i.e. in 100% of the cases), VERY FREQUENT (Present in 80–99% of the cases) FREQUENT (Present in 30–79% of the cases) OCCASIONAL (Present in 5–29% of the cases) VERY RARE (Present in 1–4% of the cases) AND EXCLUDED (Present in 0% of the cases).

Abbreviations: CM1: Chiari Malformation type 1; DD: developmental delay; ID: intellectual disability; HCC: hypoplastic corpus callosum; WV: wide ventricles.

Supplementary Table S2

Patients (N/Reference)		***cDNA***	***Microdeletions***
1	***Macchiaiolo 2022***	c.382C>T	-
2	***Macchiaiolo 2022***	c.859_860insG	-
3	***Gurrieri 2015***	c.373A>G	-
4	***Macchiaiolo 2022***	-	Microdel 19p13.2 – 134 Kb
5	***Macchiaiolo 2022***	c.95delA	-
6	***Gurrieri 2015***	c.347G>C	-
7	***Macchiaiolo 2022***	c.370_372delinsA	-
8	***Priolo 2018***	c.[28-1G >A;28-12T >A;28-13T>A]	-
9	***Macchiaiolo 2022***	c.599_602delATAG	-
10	***Macchiaiolo 2022***	c.143T>A	-
11	***Priolo 2018***	c.499C>A	-
12	***Gurrieri2015***	c.191delA	-
13	***Macchiaiolo 2022***	-	Microdel 19p13.2 - 687-793 Kb (*CACNA1A* deleted)
14	***Macchiaiolo 2022***	c.1021del	-
15	***Macchiaiolo 2022***	c.198dup	-

Supp. Table S2 **Detailed Molecular Characterization of Malan Syndrome patients (modified from Macchiaiolo et al 2022.** *J.orphanet of Rare Diseases, accepted:* DOI: 10.1186/s13023-022-02384-9)
All the variants refer to main transcript and major isoform of *NFIX* gene (NM_002501.3) (Q14938).
